# Bibliometric analysis of the scientific production on inguinal hernia surgery in the web of science

**DOI:** 10.3389/fsurg.2023.1138805

**Published:** 2023-03-22

**Authors:** Eros Ignacio Carhuallanqui-Ciocca, Johssy Yelma Echevarría-Quispe, Akram Hernández-Vásquez, Renato Díaz-Ruiz, Diego Azañedo

**Affiliations:** ^1^ Universidad Cientifica del Sur, Lima, Peru; ^2^ Centro de Excelencia en Investigaciones Económicas y Sociales en Salud, Vicerrectorado de Investigación, Universidad San Ignacio de Loyola, Lima, Peru; ^3^ Hospital III Jose Cayetano Heredia, EsSalud, Piura, Peru

**Keywords:** hernia, inguinal, hernia, abdominal, groin, general surgery, bibliometrics

## Abstract

**Objectives:**

To describe the bibliometric characteristics of the world scientific production in inguinal hernia surgery using the Web of Science Core Collection (WoS) database.

**Materials and methods:**

A search for original articles published in the WoS up to December 2021 was carried out. Bibliometric analysis was performed using the Bibliometrix data package in R and VOSviewer, version 1.6.17.

**Results:**

A total of 3,795 articles were identified in the database, with an annual average of 81 published articles and sustained growth with the highest number of publications in 2021. The article “Wide Variation and Excessive Dosage of Opioid Prescriptions for Common General Surgical Procedures” published in 2017 was the most cited (517 citations). The United States was the country of the corresponding author with the highest number of published articles (725 articles). The author with the highest number of published articles was Rosenberg J., affiliated with the University of Copenhagen, with 52 articles and 765 citations. The journal “Hernia” had the highest number of articles published on inguinal hernias in the WoS, representing 18% of the total number of articles. Finally, the keywords most used were “herniorrhaphy” and “hernioplasty” in 2008, and more recently: “single incision” “society guidelines” and “single port”.

**Conclusions:**

The scientific production of original articles on inguinal hernia surgery has increased. There is currently great interest in research on minimally invasive surgical methods and standardization of procedures based on clinical practice guidelines.

## 1. Introduction

Inguinal hernias account for more than 75% of all abdominal wall hernias, and an incidence of 13 per 10,000 population (1, 2). This abdominal wall defect causes abdominal pain and, consequently, significantly affects people's quality of life and mood, interfering with their daily and occupational activities (3). Therefore, around 20 million people worldwide undergo surgery through various techniques to repair hernia defects (4, 5). In this sense, research on inguinal hernia is necessary to obtain greater knowledge about the epidemiological aspects of the disease, as well as monitor and evaluate the efficacy and safety of health technologies aimed at its surgical management, and understand the evolution of techniques for surgical repair of hernia defects (5).

Bibliometric studies make it possible to measure and analyze the scientific production of different countries or regions and provide a general overview of certain research topics. This type of research is necessary to systematically verify and summarize this information due to the increase in the number of publications (6). Likewise, these studies show the impact and trends of scientific publications, in addition to retrospectively examining how scientific advances have been obtained and publicized (7). This information is useful for understanding the current scientific situation and guiding future research in different areas of science, including the field of surgery. In this regard, the Web of Science (WoS) Core Collection is one of the oldest databases and one of the most used in research, allowing the search for publications, analysis of citations, and other aspects and facilitating bibliometric analyses (8), since it indexes publication records of scientific journals with the greatest impact in the world. Currently, the WoS has more than 21 thousand journals, of which more than 5 thousand belong to health sciences (9).

Currently, bibliometric studies cover abdominal wall hernias; however, no bibliometric analysis of the publications on inguinal hernia, which is the most frequent condition among abdominal hernias, has been developed (10). Likewise, this condition presents particular anatomical and pathophysiological characteristics that require differentiated surgical management compared to other types of hernias, and thus, it is necessary to evaluate the bibliometric characteristics of the publications on the surgery performed in this condition (11).

The present study aimed to evaluate the bibliometric characteristics of the global scientific production of original articles on inguinal hernia surgery in the WoS Core Collection database. This analysis may be useful for professionals seeking guidance related to specialization opportunities in the field of inguinal hernia surgery and may be used for decision-making at the level of governments, universities, and research centers/groups to reorient funding and research priorities in this area.

## 2. Materials and methods

A bibliometric analysis of original articles on inguinal hernia indexed in the bibliographic database of the WoS Core Collection from inception up to May 12, 2022, was performed (9). All the publications on inguinal hernia surgery, categorized as original articles and published in WoS Core Collection up to December 2021 were included. Documents such as guidelines, consensus, errata, letters to the editor, scientific letters, editorials, corrections, retracted articles, retraction notes, and articles published in languages other than English, Spanish, or Portuguese were excluded. Only articles published until 2021 were considered because the year 2022 was still ongoing at the time of the study. The WoS Core Collection database was used as the source for this bibliometric analysis since it is a platform with access to reliable and high-quality information, and indexes records of articles of the journals with the greatest impact in the world, thereby allowing the search for articles, citation analysis and other data to facilitate bibliometric analyses (8, 9).

### 2.1. Search strategy

The following search terms were used: #1, TS = (“inguinal hernia”); #2, TS = (“groin hernia”); #3, TS = (inguinal AND hernia*); #4, TS = (groin AND hernia*); #5, #1 OR #2 OR #3 OR #4, where TS means “title and subject”; and were searched in the categories of “Surgery” and filtered to those of the type “Article”; we used the Science Citation Index Expanded (created in 1964) of WoS Core Collection (12). The search terms were proposed by one of the authors (AHV) and validated by the remaining authors, one of whom is a pediatric surgeon (RDR).

### 2.2. Selection of articles

The metadata from the full records obtained from the search were collected and downloaded as “ciw” files. Subsequently, using Rayyan (13), two authors (EICC and JYEQ) independently reviewed and selected the articles by titles, abstracts, and keywords jointly, taking into account the inclusion and exclusion criteria. Later, discrepancies were resolved jointly between the two authors (EICC and JYEQ). Next, a list was prepared, on which the WoS “Accession Number” of each record not included was recorded to repeat the search, excluding these records. After this process, the final files of the complete records were obtained for bibliometric analysis.

### 2.3. Bibliometric analysis

The bibliometric indices of the articles published for the study were obtained using the VOSviewer software and the data package Bibliometrix in R (14, 15). Also, data standardization of the data from authors, affiliations, and keywords of the records obtained was carried out manually by two authors of the present study (EICC and JYEQ) with the aim of eliminating redundancies. To do this, txt format thesauri were created, separating the data into two columns (labeled and replaced by), as recommended by the VOSviewer user manual. Subsequently, VOSviewer version 1.6.17 software was used to create co-authorship networks based on the authors, affiliations, and occurrence of retrieved records and how these change over time. Microsoft Excel was used to elaborate the corresponding tables and graphs. Network analysis was carried out using the fractional counting method, and only in the case in which the occurrence was a threshold of five mentions used for co-occurrence in titles and abstracts.

We aimed to report the following outcomes: total articles, total citations, average number of articles published annually, trends of articles and citations over the years, the ten most cited articles on inguinal hernia (including: objective of the study, author, publication year, journal, total number of citations, mean citations per year, and normalized total citations-NTC), the twenty corresponding authors countries with the highest number of published articles (including: number of articles, single country publications, multicounty publications), the ten authors with the highest production of articles on inguinal hernia (including: number of articles, total number of citations, percentage of total articles published, country and affiliation), the twenty journals with the highest number of articles published (including: number of articles, percentage of total articles, impact factor and quartile category), co-authorship network according to authors, co-authorship network according to institutional affiliations, and co-occurrence network of keywords. The Normalized Total Citations (NTC) is calculated by the Bibliometrix in R by dividing the total number of citations of an article by the average number of citations of all documents published in the same year; this measure takes into account differences in citation practices across disciplines and provides a more meaningful comparison of citation impact (16). Also, when creating the co-occurrence network of keywords, we excluded the keywords “inguinal hernia”, “hernia”, “groin” and “groin hernia” because they were the main topics of the articles in the database, and their inclusion would have made it difficult to elucidate other research topics related to the hernial condition.

In addition, the GDP per capita of the countries that are among the twenty corresponding authors countries with the highest number of published articles and the population size of each country were collected from the World Bank website (17, 18); the country's population size will be used to calculate the number of articles published per million inhabitants. Finally, we collected quartile classification data from the top twenty journals with the highest number of published articles. This data was obtained from the Journal Citation Reports website; which classifies journals from Q1 to Q4, where Q1 corresponds to “Highest ranked journals in a category” and Q4 corresponds to “Lowest ranked journals in category” (19).

### 2.4. Ethical considerations

The conduct of the study did not require the approval of an ethics committee because it was an analysis of the records of studies published in a database. The Universidad Científica del Sur approved the execution of this study than will serve as a partial requirement for two of the authors to obtain their bachelor's Degree (EICC and JYEQ).

## 3. Results

**Figure 1:**
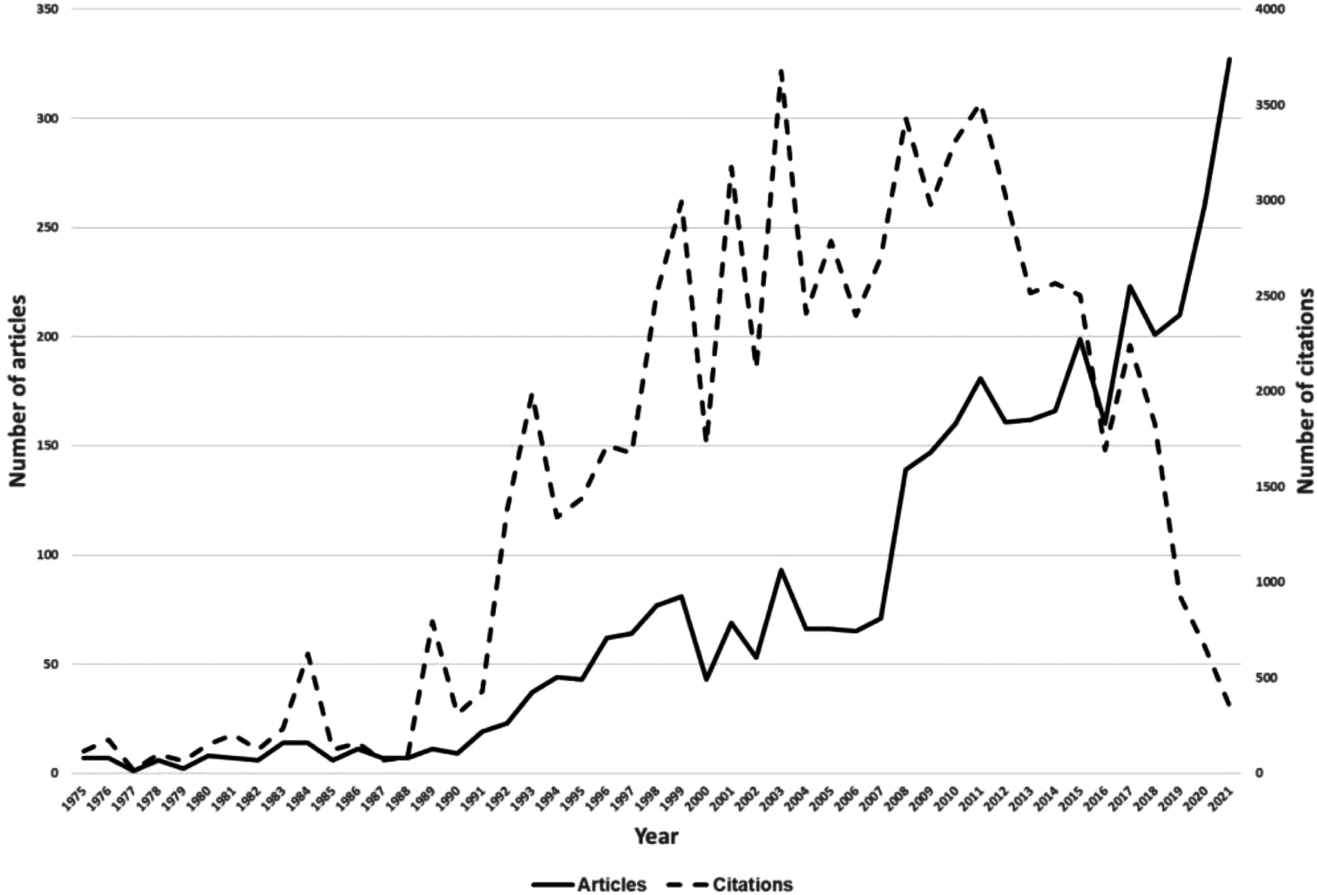
Trend of citations and articles published in web of science until 2021. The trend is represented by a polynomial trend line of degree 2.

We conducted a search in the WoS and found a total of 4,025 articles on inguinal hernia. After evaluating the titles and abstracts in Rayyan, we obtained 3,795 articles in the database, with an average of 81 articles published annually. These articles received a total of 71,349 citations. Figure 1 illustrates the trend in articles published and citations on the subject since 1975, showing that the highest number of articles published was reached in 2021, while the highest number of citations was observed in 2003.

The 10 most cited articles on inguinal hernia in the WoS had a total citation range between 270 and 517. The article with the highest number of citations (517) was titled “Wide Variation and Excessive Dosage of Opioid Prescriptions for Common General Surgical Procedures” (20), published in Annals of Surgery in 2017. The other articles in the top 10 described diverse topics, such as the biochemical aspects of inguinal hernias, surgical techniques, and complications (Table 1).

**Table 1:** The ten most cited articles on inguinal hernia in the Web of science.

Title	Objective of the study	Authors	Year of publication	Journal	Total Number of citations	Mean citations per year	NTC
Wide Variation and Excessive Dosage of Opioid Prescriptions for Common General Surgical Procedures	Examine opioid prescription patterns after general surgery procedures and estimate an ideal number of pills to prescribe.	Hill MV et al.	2017	Annals of Surgery	517	103	51
The treatment of complicated groin and incisional hernias	Discuss some alternatives for surgeons, mostly technical considerations, and only in adults for the management of complicated and incisional hernias	Stoppa RE et al.	1989	World Journal of Surgery	497	15	7
Systemic cytokine response after major surgery	Explore the association between plasma cytokine levels, clinical course, and C-reactive protein in major surgery	Baigrie RJ et al.	1992	British Journal of Surgery	487	16	8
Demographic and socioeconomic aspects of hernia repair in the United States in 2003	Describe the demographic and socioeconomic aspects of abdominal wall hernia repair in the US	Rutkow LM et al.	2003	Surgical Clinics of North America	435	23	11
Laparoscopic repair of incisional abdominal hernias using expanded polytetrafluoroethylene: preliminary findings	To describe the preliminary findings of the use of polytetrafluoroethylene in the repair of incisional abdominal hernias in 5 cases	Leblanc KA et al.	1993	Surgical Laparoscopy Endoscopy & Percutaneous Techniques	391	13	7
Pain and functional impairment 1 year after inguinal herniorrhaphy: a nationwide questionnaire study	To determine the incidence of groin pain 1 year after inguinal herniorrhaphy and assess the influence of chronic groin pain on functionality	Bay- Nielsen M et al.	2001	Annals of Surgery	388	18	8
Feasibility of robotic laparoscopic surgery: 146 cases	Evaluate the feasibility of robotic laparoscopic surgery.	Cadiere GB et al.	2001	World Journal of Surgery	301	14	7
Foreign body reaction to meshes used for the repair of abdominal wall hernias	To investigate local tissue reactions to meshes removed from humans.	Klinge U et al.	1999	European Journal of Surgery	293	13	8
Prospective study of chronic pain after groin hernia repair	To investigate chronic pain in patients 1 year after surgery	Callesen T et al.	1999	British Journal of Surgery	279	12	8
Laparoscopic inguinal herniorrhaphy. Results of. Multicenter trial	To determine if laparoscopic inguinal herniorrhaphy represents a viable alternative to conventional repair	Fitzgibbons R et al.	1995	Annals of Surgery	270	10	8

TAPP, transabdominal preperitoneal patch plasty; TEP, total extraperitoneal patch plasty; NTC, normalized total citations.

NTC = (total number of citations/average *n*° of citations of all documents published in the same years).

The results are based on the search carried out for articles until December 2021.

The United States had the highest number of published articles with a corresponding author affiliated with the country, with 725 articles, followed by the United Kingdom and China, with 284 and 280 articles. In terms of publications with authors from the same country, the United States led with 673, followed by China with 265 and the United Kingdom with 261. In relation to multiple country publications, Brazil led with 92 articles, followed by United States with 52, and Italy with 26. Table 2 also provides the gross domestic product per capita of the corresponding author countries, with the highest GDP per capita of $ 61 dollars in the United States, followed by $ 60 dollars in Denmark and $ 56 dollars in Australia. On the other hand, the country with the highest number of articles published per million inhabitants is Denmark, with 16.8 articles, followed by Sweden and the Netherlands (Table 2).

**Table 2:** The twenty corresponding author countries with the highest number of published articles.

Country	No. of articles	SCP	MCP	GDP per capita ($)^a^	Population in 2021(millions)^b^	Number of articles published per million inhabitants
United States	725	673	52	61	331.9	2.2
United Kingdom	284	261	24	42	67.3	4.2
China	280	265	15	11	1,412	0.2
Germany	206	169	39	45	83.2	2.5
Japan	204	202	2	35	125.7	1.6
Turkey	196	195	1	8	84.8	2.3
Italy	179	153	26	31	59.1	3
India	164	163	1	2	1,408	0.1
Netherlands	149	142	11	51	17.5	8.5
Sweden	122	106	16	54	10.4	11.7
Denmark	99	90	9	60	5.9	16.8
Spain	72	65	7	27	47.4	1.5
Australia	71	69	2	56	25.7	2.8
France	60	53	7	38	67.8	0.9
Korea	60	58	2	31	51.7	1.2
Egypt	53	50	3	4	109.3	0.5
Belgium	52	43	9	46	11.6	4.5
Canada	51	42	9	46	38.3	1.3
Greece	49	43	6	18	10.6	4.6
Brazil	46	37	92	9	214.3	0.2

SCP, Single country publications; MCP, multiple country publications; GDP, gross domestic product ($): US dollar.

^a^ Collected from: https://data.worldbank.org/indicator/NY.GDP.PCAP.CD.

^b^ Collected from: https://datos.bancomundial.org/indicator/SP.POP.TOTL.

**Table 3:** The ten authors with the highest production of articles on inguinal hernia in the Web of science.

Author	Number of articles	Total number of citations	% of total publications (*n* = 3 795)	Country	Affiliation
Rosenberg J	52	765	1.4	Denmark	University of Copenhagen
Kockerling F	44	1015	1.2	Germany	Vivantes Hospital
Nordin *P*	42	1636	1.1	Sweden	Umea University
Bittner R	38	1370	1	Germany	Ulm University
Andresen K	30	275	0.8	Denmark	Herlev Hospital
Kehlet H	30	1747	0.8	Denmark	Rigshospitalet
Sandblom G	28	932	0.7	Sweden	Karolinska Institute
Schumpelick V	26	1851	0.7	Germany	RWTH Aachen University
Lange JF	24	718	0.6	Netherlands	Erasmus University
Klinge U	23	1844	0.6	Germany	RWTH Aachen University

**Figure 2:**
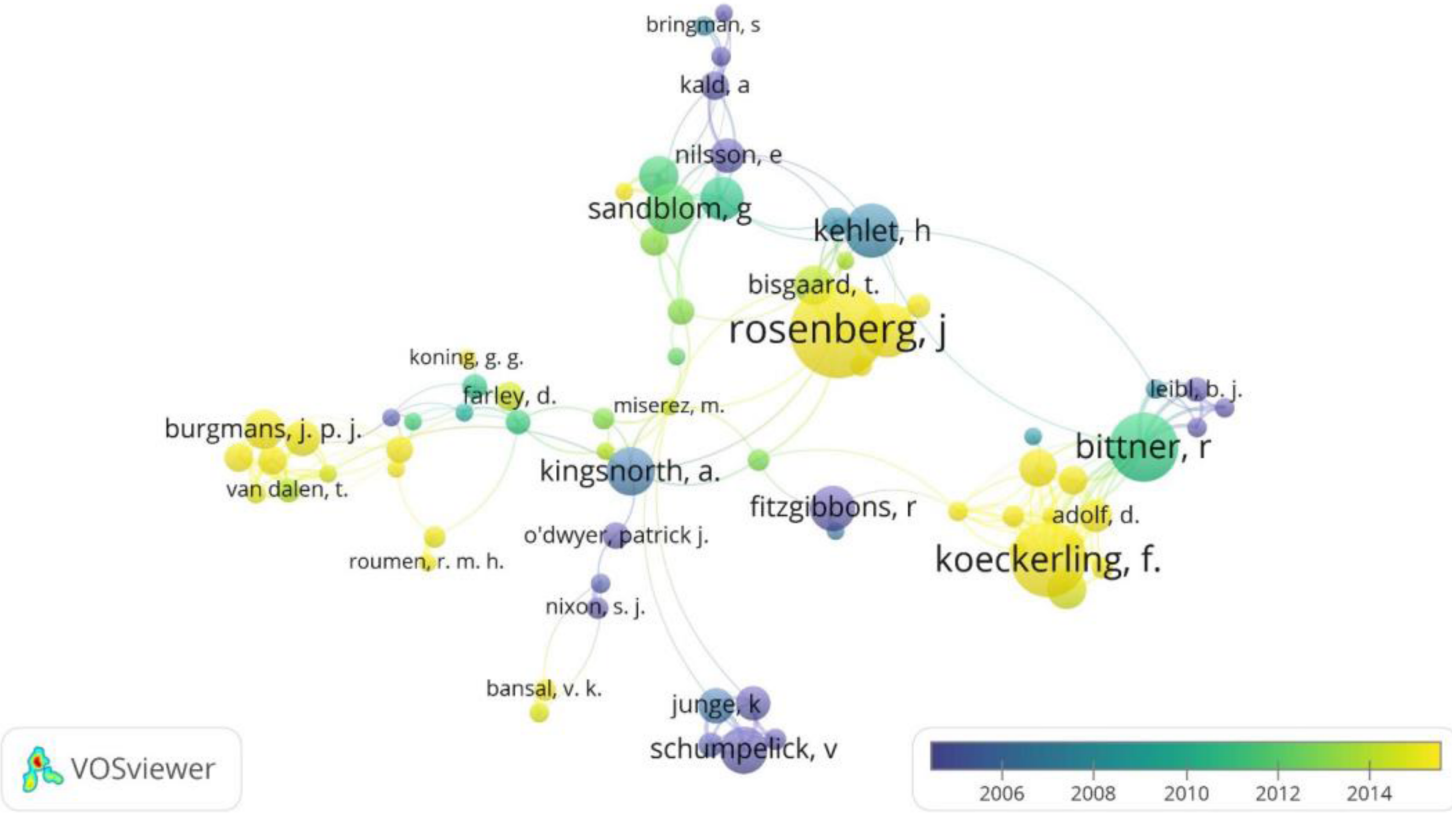
Bibliographic coupling network of authors with the greatest number of articles published in the web of science database.

Rosenberg J., affiliated with the University of Copenhagen, had the highest production of articles on inguinal hernia, with a total of 52 published articles and 765 citations, followed by Kockerling F. (44 articles and 1,015 citations), and Nordin P. (42 articles and 1,636 citations) (Table 3). The top 10 authors with the most publications on inguinal hernia were affiliated with Denmark, Germany, Sweden, and Netherlands. Among them, Bittner, R and Sandblom, G, had a higher publication frequency around 2010. In addition, Amid and Kald presented the greatest scientific production on the subject in the 2000s (Figure 2).

*Hernia* had the highest number of articles published on inguinal hernia in the WoS, with 687 articles, representing 18% of the total articles included in the analysis. *Surgical Endoscopy and other Interventional Techniques* was in second place with 351 articles (9% of the total) followed by the *Journal of Laparoendoscopic & Advanced Surgical Techniques* with 173 articles (5% of the total). *Annals of Surgery* had the highest impact factor of 12.96, as compiled from *Journal Citation Reports* in Clarivate*,* followed by the *British Journal of Surgery* with 6.94 and the *Journal of The American College of Surgeons* with 6.113. According to the WoS classification, most journals were Q1, 4 belonged to Q2, 5 to Q3, and 3 to Q4, (Table 4).

**Table 4:** The twenty journals with the highest number of articles published on inguinal hernia in the web of science.

Journal	No. of articles	Percentage of total articles (*n* = 3 795)	Impact Factor^a^	Quartile Category^b^
Hernia	687	18	4.739	Q1
Surgical Endoscopy and other Interventional Techniques	351	9	4.584	Q1
Journal of Laparoendoscopic & Advanced Surgical Techniques	173	5	1.878	Q3
British Journal of Surgery	140	4	6.939	Q1
World Journal of Surgery	139	4	3.352	Q2
American Journal of Surgery	136	4	2.565	Q2
American Surgeon	120	3	0.688	Q4
Surgical Laparoscopy Endoscopy & Percutaneous Techniques	109	3	1.719	Q3
Annals of Surgery	98	3	12.969	Q1
International Journal of Surgery Case Reports	85	2	NA	NA
International Surgery	77	2	0.056	Q4
Surgery	75	2	3.982	Q1
Surgery Today	72	2	2.549	Q2
Journal of The American College of Surgeons	67	2	6.113	Q1
Surgical Endoscopy-ultrasound and Interventional Techniques	65	2	4.584	Q1
Surgical Clinics Of North America	61	2	2.741	Q2
Annals of The Royal College of Surgeons of England	60	2	1.891	Q3
Indian Journal of Surgery	60	2	0.656	Q4
Journal of Surgical Research	58	2	2.192	Q3
BMC Surgery	56	2	2.102	Q3

^a^ Collected from Journal Citation Reports 2020. NA (Not available).

^b^ Quartile rankings based on rank for the Journal Impact Factor. In Journal Citation Reports, quartiles are defined as the following: Q1 (Highest ranked journals in a category), Q2, Q3 and Q4 (Lowest ranked journals in a category). For more details see: https://support.clarivate.com/ScientificandAcademicResearch/s/article/Journal-Citation-Reports-Quartile-rankings-and-other-metrics?language=en_US.

**Figure 3:**
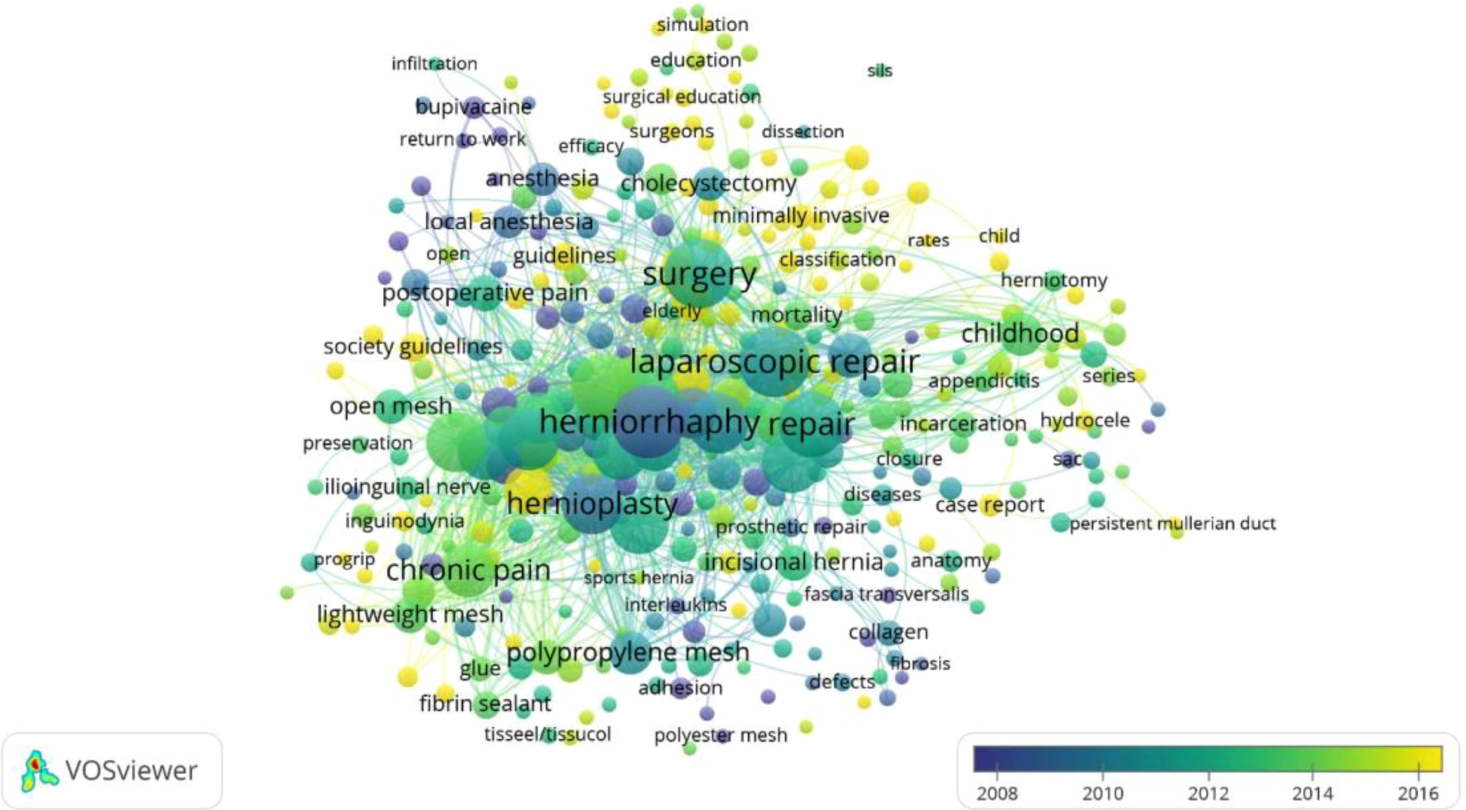
Bibliographic coupling network of terms most used in the web of science database.

In the network of co-occurrence of the most used keywords in the WoS Core Collection database, the terms “herniorrhaphy” and “hernioplasty” were the most frequently used in 2008. In the following years, new terms have been used in research like “laparoscopic repair” and “surgery” having a higher frequency of use between 2010 and 2012. Finally, in 2016, the use of terms such as “society guidelines”, “single incision”, and “single port”, among others, increased. Additionally, the terms “simulation” and “surgical education” started to be used from the middle of 2014 onwards (Figure 3).

**Figure 4:**
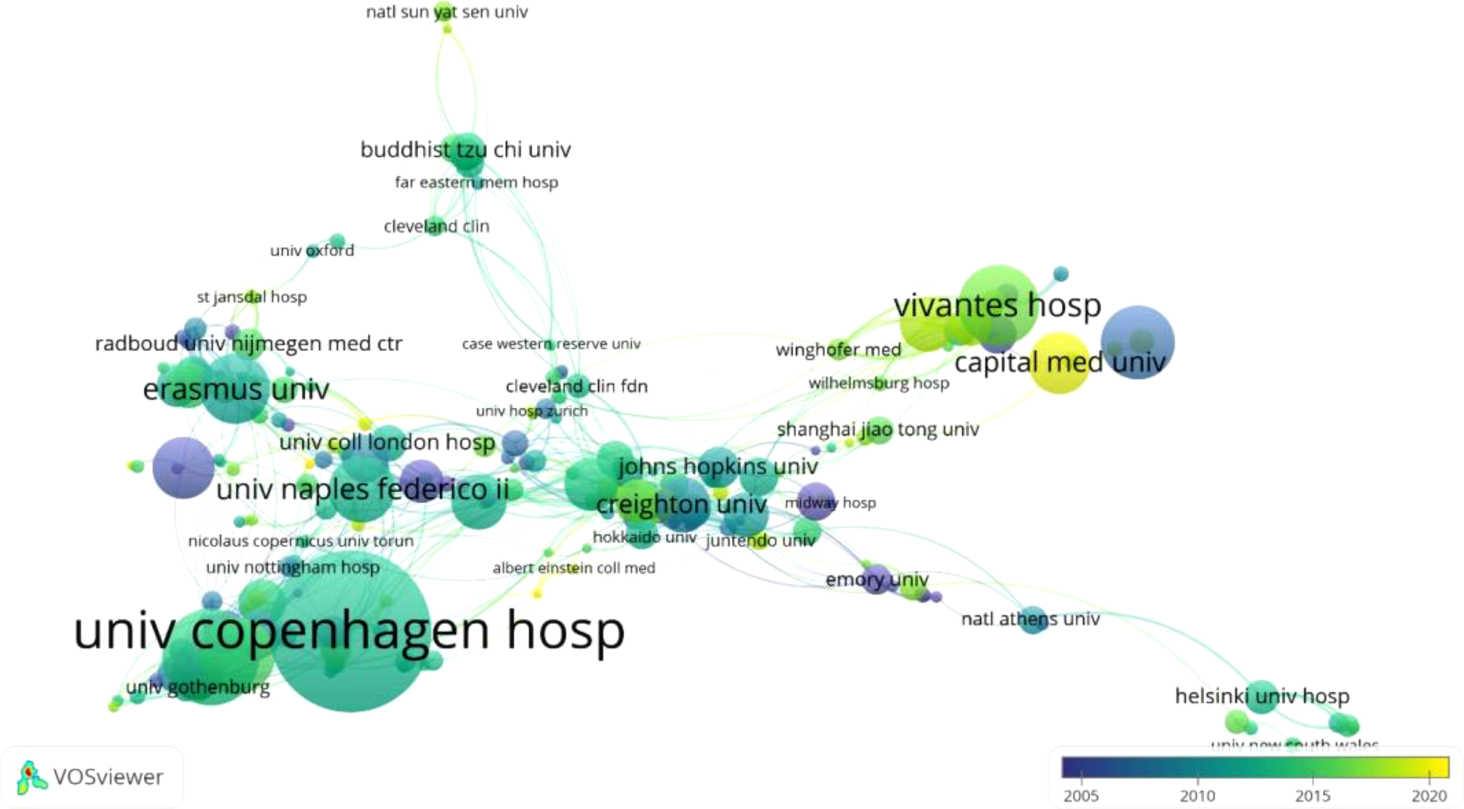
Bibliographic coupling network of institutions with the highest scientific production in the web of science database.

In the cluster analysis map regarding the institutional affiliations that produced literature on hernia surgery (Figure 4, “univ Copenhagen hosp” had the highest scientific production on the subject from 2012 to 2014. “Johns Hopkins univ,” “Erasmus univ” and “Creighton univ” presented a considerable level of literature production on the subject during the same period. Since 2016, the “Capital med univ” has been leading production followed by the “German red cross hosp”.

## 4. Discussion

The study aimed to evaluate the bibliometric characteristics of the global scientific production of original articles on inguinal hernia surgery in the WoS Core Collection database until December 2021. The analysis revealed a continuous growth in the scientific production on this topic, from 1990 to the search date, in which Rosenberg J. had the highest production of articles on inguinal hernia with 52 publications, representing 1.4% of all included publications. The most cited article was “Wide Variation and Excessive Dosage of Opioid Prescriptions for Common General Surgical Procedures” (20) with an average of 103 citations per year. This article discusses the variation in opioid prescription to reduce postoperative pain in different surgeries, including open and laparoscopic inguinal hernia surgeries. The remaining articles among the ten most cited, were published more than 15 years ago, with the second most cited article dating back to 1989, which describes the management of complications of inguinal and incisional hernias (21); other articles focus on demographic characteristics, pathophysiology, use of mesh, and operative techniques. This absence of highly cited recent articles may be attributed to the impact of these older articles have had in shaping current knowledge on hernia management and treatment.

Rosenberg, J, a is a widely recognized Danish author, who has produced the highest number of articles on inguinal hernias, totaling 54 articles and 765 citations, with publications on postoperative pain, systematic reviews on surgical approaches, complications, among others. Kockerling F., a German author, follows Rosenberg with more than 1,000 citations in 44 articles published on similar topics. Since 2015, Rosenberg and Kockerlingśs publications have gained more prominence, with a focus on generating evidence synthesis for decision-making (5, 22–24) in the management of various types of hernia in clinical practice. Recent publications have also aimed to discover new approaches to treat inguinal hernias, use of minimally invasive methods, considerations in the postoperative period, the development of practice guidelines, reducing possible complications, and their association with other system pathologies or conditions (25–27).

The United States had the most publications in our analysis, likely due to its high investment in research and development, which reaches 3.45% of the GDP per capita (28). Factors such as high GDP per capita, population size, government investment in tertiary education, and gross domestic spending, contribute to a countrýs scientific and socioeconomic development (28, 29). However, when we consider the number of articles published per million inhabitants, we found that Denmark, Sweden and the Netherlands emerge as leaders in this aspect. It is noteworthy as these countries are also among the countries of origin of the top ten authors with the highest production on inguinal hernia; this suggests that these countries have a greater capacity to generate new knowledge in the topic despite having a smaller population size compared to other countries. On the other hand, the journal *Hernia* had the highest number of articles published on inguinal hernia in the WoS Core Collection, 687 articles (18% of total), followed by S*urgical Endoscopy and Other Interventional Techniques (*9% the total), both based in the United States (30, 31). These journals focus on specialized surgery topics, and therefore, resulting in lower impact factor compared to broader scope journals like Annals of Surgery, which have higher citation opportunities (32).

Regarding the co-occurrence network of terms in the WoS Core Collection database, “herniorrhaphy” and “hernioplasty” were initially the most commonly used terms, both referring to open surgical approaches involving incisions and suturing layers of tissue or using a mesh to correct hernial defects (33). Between 2010 and 2012, “laparoscopic repair” became more frequent, reflecting its current status as the standard management for uncomplicated hernias worldwide. The two most commonly used laparoscopic techniques are laparoscopic transabdominal peritoneal and transabdominal preperitoneal repair, which have comparable outcome in terms of hernia recurrence and chronic pain (33, 34). In 2014, “chronic pain” emerged as a frequent keyword, indicating a shift towards patient-reported outcomes and long-term complications of old surgical techniques (34). The use of terminology such as “society guidelines” “increase in 2016, reflecting the growing interest in synthesizing research evidence on hernia management to develop evidence-based recommendations through clinical practice guidelines. Despite excluding the guidelines from the final database in our study, the term still appeared as a keyword; this may respond to the fact that some of the articles in the database had the objective of evaluating surgeons” compliance with clinical practice guidelines (35, 36). Recent articles have also focused on “simulation”, which is a training tool for surgeons, with studies such as “Simulated training model in a low cost for laparoscopic inguinal hernioplasty” (37) demonstrating improved surgical skills through simulation-based training.

Among the limitations of the study, the total number of citations of an article does not necessarily reflect its impact on disease management. The duration during which citations are accumulated is also an important factor to consider since recent articles may not yet have enough citations to surpass older publications. Furthermore, the search was only conducted in WoS, which may exclude studies published in other databases, such as Scopus or MEDLINE. However, WoS offers a variety of metrics and indicators beyond the impact factor, allowing for a more comprehensive and unbiased analysis of the journals and the quality of the information. WoS also provided vast information which has allowed the standardization and updating of these topics.

In conclusion, the evolution of research on inguinal hernia surgery in recent years is significant, with a shift from focusing on early surgical techniques to the development of new technologies and minimally invasive methods, as well as the creation of clinical practice guidelines. Knowledge of current trends in research in inguinal hernia surgery can be useful for the adoption and proposal of new lines of research, at the level of universities and research centers/groups. Likewise, these results can guide budgetary priorities for government agencies and industry to finance the development of new techniques, materials, and devices that seek to optimize the surgical management of inguinal hernias.

## Data Availability

The original contributions presented in the study are included in the article/supplementary material, further inquiries can be directed to the corresponding author/s.
